# Tunability of Photovoltaic Functions via Halogen Substitution [(Ade)_2_ CdX_4_](X = Cl, Br): A Class of Three-Dimensional Organic–Inorganic Hybrid Materials

**DOI:** 10.3390/molecules29122773

**Published:** 2024-06-11

**Authors:** Meixia Lv, Hongzhi Hu, Abuduheni Adila, Yibo Yan, Yang Liu, Zunqi Liu

**Affiliations:** 1 Chemistry and Chemical Engineering College, Xinjiang Agricultural University, Urumqi 830052, China; lvmeixia1105@163.com (M.L.); huhongzhi305@163.com (H.H.); 17799751675@163.com (A.A.); yanyibo2022@163.com (Y.Y.); 2 Xinjiang Sub-Center National Engineering Research Center of Novel Equipment for Polymer Processing, Urumqi 830052, China; 3 Xinjiang Key Laboratory of Agricultural Chemistry and Biomaterials, Urumqi 830052, China

**Keywords:** metal halide, three-dimensional framework, photoluminescence, dielectric, electrochemical

## Abstract

Two new three-dimensional organic–inorganic hybrid crystalline materials, [(Ade)_2_ CdCl_4_] (**1**) and [(Ade)_2_ CdBr_4_] (**2**), were obtained by the slow evaporation of adenine (Ade) and cadmium chloride in aqueous solution at room temperature with hydrochloric acid and hydrobromic acid used as halogen sources. The structural, thermal, optical, and electrical properties were characterized by single-crystal X-ray diffraction, infrared spectroscopy, thermogravimetric analysis, variable-temperature–variable-frequency dielectric constant analysis, and electrochemical tests. With increasing the substitution of Cl by Br, the composition of the material changed and the space group shifted from *P*-1 to *P*2_1_/m, with a significant blue-shift in the fluorescence emission. Changing the temperature induced the deformation of the three-dimensional framework structure formed by hydrogen bonding interactions, leading to dielectric anomalies. Cyclic voltammetry tests showed the good reversibility of the electrolysis process. The structural diversity of the complexes was realized by modulating the halogen composition, and a new method for designing novel organic–inorganic hybrids with controllable photoelectric functionality was proposed.

## 1. Introduction

Organic–inorganic hybrid multifunctional materials that exhibit phase transition, photoluminescence (PL), magnetism, dielectricity, and ferroelectricity can be formed through the self-assembly of organic cations and inorganic anions. These materials have a wide range of applications in sensing, data storage, and light-emitting diodes [1,2,3,4,5,6,7]. Introducing nitrogen-containing organic small molecules into hybridized materials can lead to structurally variable PL properties, which have attracted the attention of researchers [8,9,10,11]. Adenine (C_5_H_5_N_2_) is a rigid nucleic base containing four N atoms and one NH_2_ group [12,13,14,15]. Adenine has many potential structural modes, and it can bond with metal ions to form a variety of coordination modes and can also self-assemble into supramolecular structures by taking advantage of the Watson–Crick, Hoogsteen, and other weak hydrogen bonding interactions that are unique to the nucleic acid base [16,17,18,19,20,21]. The binding sites in monodentate ligands are usually N3, N7, and N9. Porous crystalline materials can be constructed with zero-dimensional, one-dimensional, two-dimensional, or three-dimensional topologies and novel functions [22,23,24,25,26,27].

Large bandgaps, highly localized charge distributions with very high exciton binding energies, and stable exciton emission energies can be achieved for cadmium metal ions in low-dimensional organic–inorganic hybrid materials [28,29,30,31,32]. Zou et al. [33] reported a new hybridized metal halide for application as a solid-state light source, namely, [C_6_H_7_ClN]CdCl_3_, where C_6_H_7_ClN = 4-chloromethylpyridine cation, in which the CdCl_6_ octahedron forms a corrugated 1D double-chain structure by sharing non-coplanar edges, and it exhibits white-light emission with a photoluminescence quantum yield (PLQY) of 12.3% under 345 nm UV excitation. Introducing the transition metal Cd is a new approach for manipulating the luminescent properties of hybrid materials.

Structural control of metal halide anions is a promising approach for modulating the optoelectronic properties of complexes [34,35,36]. Fu et al. [37]. synthesized three zero-dimensional (0-D) organic–inorganic hybrid functional materials, [BMPD]ZnX_4_ (BMPD = 1-butyl-1-methylpiperidinium cation, X = I, Br, and Cl), where the *T_c_* (*T_c_ =* phase transition temperature) gradually increased from 330 K to 344 K with the sequential substitution of the halogen atoms from I to Cl. A reversible dielectric anomaly was apparent during the heating and cooling processes. The BMPD-Br material emits blue light, attributed to self-trapping exciton emission due to strong electron–phonon coupling in the lattice.

The transition metals zinc and cadmium are in the same main group (IIB) on the periodic table and have completely filled d orbitals and two s electrons in the outermost shell. Compared with zinc, cadmium (a metal element) has a richer coordination behavior and more geometrical configurations; moreover, cadmium complexes have excellent optical properties [29,38,39,40,41,42,43,44,45,46,47,48,49]. To further investigate the structure and properties of adenine and cadmium complexes, in this study, we designed and synthesized two new three-dimensional organic–inorganic hybrid crystalline materials, namely, [(C_5_H_6_N_5_)_2_ CdCl_4_] (**1**), with the abbreviated formula [Ade-Cl], and [(C_5_H_6_N_5_)_2_ CdBr_4_] (**2**) with the abbreviated formula [Ade-Br]. The compositions, single-crystal structures, and thermal, electrical, and photoluminescent properties of the complexes were characterized by infrared spectroscopy, single-crystal and powder X-ray diffraction (XRD) techniques, thermogravimetric analysis (TGA), variable-temperature–variable-frequency dielectric constant analysis, solid-state fluorescence testing, and cyclic voltammetry analysis.

## 2. Results and Discussion

### 2.1. Single-Crystal Structures of Compounds ***1*** and ***2***

The structures of Ade-Cl and Ade-Br were determined at 100 K and 293 K. With increasing the temperature, the *a*, *b*, and *c* cell parameters of Ade-Cl increased by 0.77%, 0.95%, and 0.69%, while *α, β*, *γ*, and the volume (*V*) increased by 0.63%, 0.36%, 0.39%, and 2.06%, respectively (Table 1). With the substitution of Br atoms for Cl atoms, the space group changed from *P-1* for Ade-Cl to *P*2_1_/m for Ade-Br, and with increasing in temperature, the *a*, *b*, and *c* cell parameters of Ade-Br increased by 0.71%, 0.64%, and 0.55%, while *β* and *V* increased by 0.25% and 1.99%, respectively, which is more significant compared to the change in the cell parameters of Ade-Cl.

**Figure 1:**
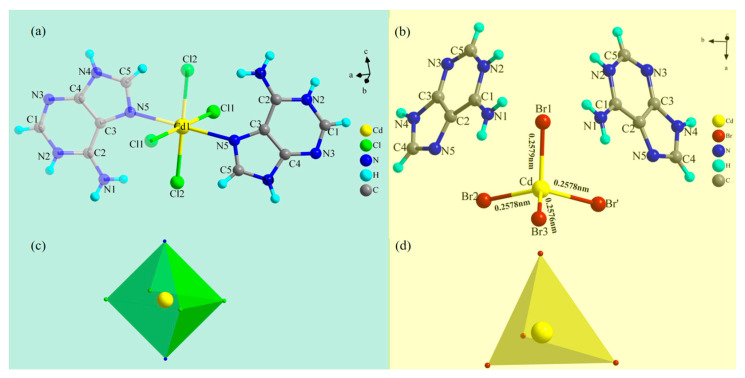
Simplest structures of Ade-Cl (**a**) and Ade-Br (**b**) at 293 K; polyhedrons of Ade-Cl (**c**) and Ade-Br (**d**).

Figure 1a shows the symmetrical structure of Ade-Cl at 293 K, where the cadmium center interacts with four chlorine atoms and two protonated adenine cations molecules through Cd-Cl and Cd-N coordination bonds, forming an octahedral structure (Figure S1). Figure 1c shows Ade-Cl in a six-coordinate octahedral configuration, where the range of distances from the central atom, Cd, to the octahedral vertices at 293 K was 0.2569–0.2570 nm, the average bond length was 0.2569 nm, and the Cd-N bond length was 0.2508. The stable octahedral configuration formed by Cl-Cd-Cl had an average bond angle of 88.553°, which was smaller than that of N-Cd-Cl, with a bond angle of 90.518° (Table S1). At low temperatures, the bond lengths and bond angles of the octahedron changed to some extent. With increasing the temperature, the distance from the center atom to the vertex increased by 0.23%, the Cd-N length decreased by 0.12%, and the average bond angle increased by 0.51%. The bond lengths and bond angles underwent telescopic deformations with the change in temperature, leading to a change in the arrangement of Ade-Cl within the spatial structure. Figure 1b shows the simplest structure of Ade-Br at room temperature, comprising one tetrahedral [CdBr_4_]^2−^ anion and two protonated [Ade]^+^ cations. Figure 1d shows the tetrahedral configuration of the [CdBr_4_]^2−^ anion of Ade-Br. The Cd-Br bond lengths were in the range of 0.2576–0.2579 nm at 100 K, with an average bond length of 0.2579 nm, and the Br-Cd-Br bond angles were in the range of 104.34–110.97°, with an average angle of 107.67°; the bond lengths and angles at 293 K are summarized in Table S2. Within the Ade-Cl structure, cadmium and the adenine molecule form a unit through the Cd-N coordination bond, but because the radius of the bromine atom is larger than that of the chlorine atom, the [Ade]^+^ cation and the [CdBr_4_]^2−^ anion cannot form a metallic coordination bond, and ionic complexes are thus formed. Structural analysis shows that the Cd-Cl bond length is shorter than the Cd-Br bond length at the same temperature, and the Cl-Cd-Cl bond angle is also smaller than the Br-Cd-Br bond angle. The difference in the bond lengths and angles leads to structural differences, where the change in the structure of Ade-Cl and Ade-Br is expected to lead to differences in the optoelectronic properties.

**Figure 2:**
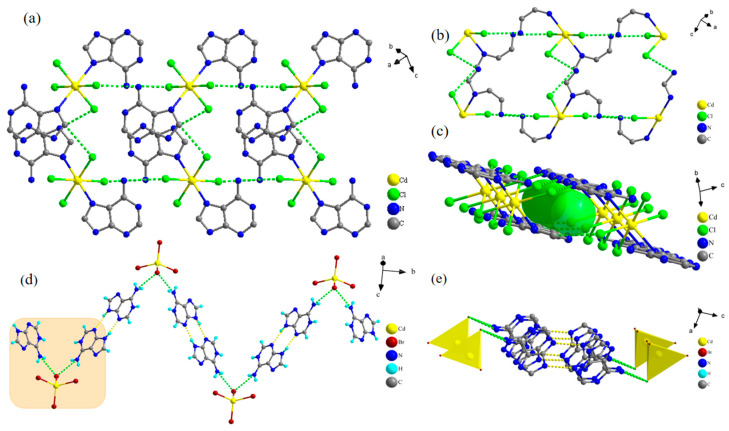
One-dimensional chain structure of Ade-Cl (**a**), remove excess atomic cavity structure (**b**), sub-atomic channel diagram (**c**), one-dimensional chain structure of Ade-Br (**d**), one-dimensional chain diagram of the ac plane (**e**).

The arrangement of the components within the spatial structure of a material will directly affect the physical properties of the material. Among the non-classical hydrogen bonding interactions, the C-H···Cl interaction has attracted interest in the broad field of materials chemistry [50,51]. At low temperatures, the Ade-Cl molecules interact with each other via N-H···Cl and C-H···Cl hydrogen bonds, forming an infinite one-dimensional chain (Figure 2a and Figure S2). By removing redundant atoms, the spatial arrangement with certain structural features becomes apparent (Figure 2b). The introduction of subatoms between two cadmium atoms after connecting the hydrogen bonds forms an ion channel (Figure 2c). At 100 K, the N-H···Cl hydrogen bond lengths were in the range of 0.31369–0.33070 nm, with an average bond length of 0.31877 nm, and the bond angle was in the range of 145.349–171.678°, with an average bond angle of 159.372°; the C-H···Cl hydrogen bond length was in the 0.33736–0.34337 nm range, with an average bond length of 0.340365 nm, and the bond angles were in the 145.349–171.678° range, with an average bond angle of 111.344° (Table S3). With increasing the temperature, the average bond length of N-H···Cl increased by 0.49% and the average bond angle increased by 0.146%; the average bond length of C-H···Cl increased by 1.20% and the average bond angle increased by 1.10%. The telescopic deformation and torsion arising from the hydrogen bond lengths and bond angles cause elongation within the Ade-Cl one-dimensional chain structure. To investigate the difference between the one-dimensional chain structure of Ade-Br and Ade-Cl after the substitution of bromine atoms for chlorine atoms, two molecules of protonated adenine cations and [CdBr_4_]^2−^ anions were used to form “ripple” chain diagrams on the *bc* side (Figure 2d and Figure S3) and on the *ac* side (Figure 2e) through the interaction of the N-H···Br and N-H···N hydrogen bonds, as well as chain diagrams on the *ac* side (Figure 2e). The comparison shown in Table S4 reveals that the hydrogen bond length increased by 0.49% and the bond angle increased by 0.35% when the temperature was increased from 100 K to 293 K. The changes in the bond length and bond angle cause stretching and twisting in Ade-Br, ultimately leading to the elongation of the chain structure.

In the complexes Ade-Cl and Ade-Br, the metal cadmium is the central atom and is not occupied by the ligand vacancy metal, and the metal and chlorine form coordination bonds and eventually form antibonding vacant orbitals. NH bonds between the electrons are filled to the antibonding orbitals to form a feedback bond, leading to the formation of a stable hydrogen bonding structure.

**Figure 3:**
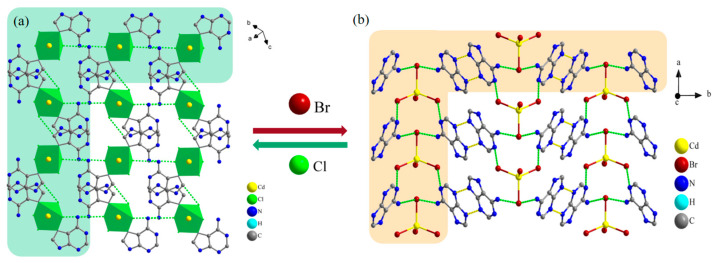
Diagram of hydrogen bonding network in 2D Ade-Cl (**a**) and 2D Ade-Br (**b**).

The one-dimensional chain structure of Ade-Cl forms a two-dimensional network in the *bc* plane through hydrogen bonding between the chains, as shown in Figure 3a. The Ade-Br chains interact with each other through π-π interactions, presenting an ‘organic layer-metal complex-organic layer’ stacking arrangement, forming an infinite derivative two-dimensional network in the *ab* plane in a ‘sandwich’ structure, as shown in Figure 3b.

**Figure 4:**
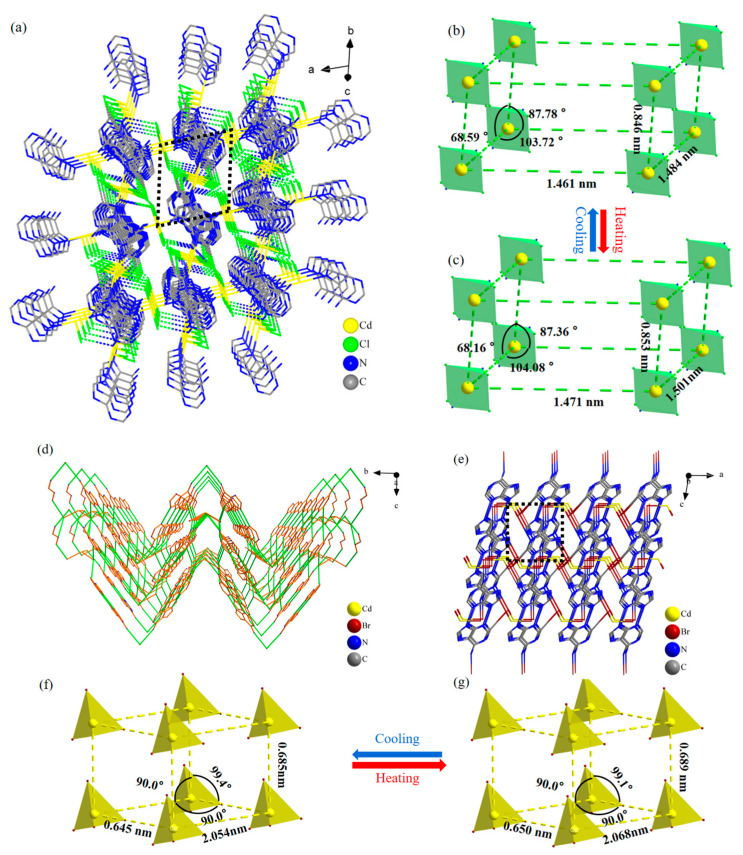
Ade-Cl 3D framework diagram (**a**); hexahedral diagrams with cadmium as vertex (**b**,**c**); Ade-Br 3D framework diagrams (**d**,**e**); hexahedral diagrams with cadmium as vertex (**f**,**g**).

The two-dimensional mesh layers formed in the space of the two compounds generate a three-dimensional framework structure under the action of hydrogen bonding, as shown in Figure 4. Figure 4a shows the three-dimensional framework diagram of Ade-Cl, with adenine molecules connected between the layers of the inorganic metal framework, which are neatly aligned in the *ac* plane. To clearly represent the changes in the spatial structure, redundant atoms were deleted to obtain the cadmium atoms as the vertices of the three-dimensional framework, as shown in Figure 4b,c. With the change in the temperature of Ade-Cl, the lengths between the three vertices of the cadmium atoms and the angle between the three vertices changed significantly.

Figure 4d shows the three-dimensional framework of Ade-Br after deleting the redundant atoms, which is neatly arranged in the *bc* plane in the shape of “ripples” (Figure S4). To explore the changes in the three-dimensional framework of the cadmium atom as the apex within the spatial structure and to select the appropriate metal cadmium ions in the *ac* plane, the three-dimensional metal ion framework is presented in Figure 4f,g. For Ade-Br, the two intercalation angles were unchanged, whereas the interatomic length changed slightly. With temperature variation, the bond length, bond angle, and angle between the neighboring faces changed, leading to the deformation of the three-dimensional structure. The holes were also subjected to a certain degree of stretching and twisting at different temperatures, ultimately leading to a change in the optoelectronic properties of the material.

From the above structural analysis, it is not difficult to see that the chemical composition of the two crystalline materials, Ade-Cl and Ade-Br, changed due to halogen substitution from Cl to Br. The space group changed from *P*-1 to *P*2_1_/m, and the coordination mode of the cadmium atoms changed considerably. The Cd in Ade-Cl is six-coordinate with a sp^3^d^2^ orbital hybridization and an octahedral structure. In addition to the Cd-Cl bond, cadmium interacts with the nitrogen atoms on adenine to form Cd-N coordination bonds, whereas the Cd in Ade-Br is four-coordinate and sp^3^-orbital-hybridized with a tetrahedral configuration (all with Cd-Br bonds). As indicated by the one-, two-, and three-dimensional spatial arrangements, the two compounds are arranged differently. Under the influence of temperature, the bond lengths, hydrogen bonds, and bond angles of Ade-Cl and Ade-Br changed significantly, ultimately leading to telescopic deformations and torsional changes in the framework structure, resulting in controllable alterations in the thermal, electrical, and optical properties.

### 2.2. Hirshfeld Surface Analyses of Compounds ***1*** and ***2***

**Figure 5:**
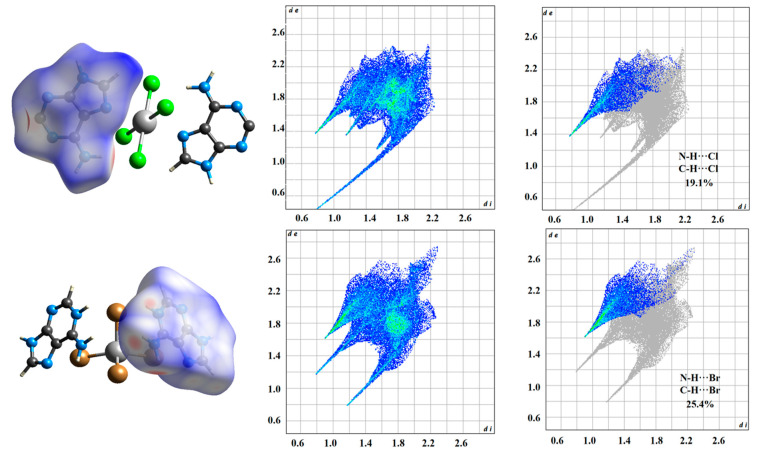
Hirshfeld surfaces and 2D fingerprint plots of Ade-Cl and Ade-Br.

Hirshfeld surface analysis is an intuitive tool to observe the distribution of intermolecular interactions. Surface features reflect interactions between different atomic sizes, as well as intermolecular contact distances, thus reflecting intermolecular interactions, and the corresponding 2D fingerprint plots were carried out and analyzed, as shown in Figure 5.

In Ade-Cl, the intermolecular interactions between the carbon, nitrogen, and H atoms on the adenine molecule appear as red regions on the Hirshfeld surface, with bright red spots corresponding to C-H···Cl and N-H···Cl interactions, intermolecular interactions in 2D fingerprinting. In C-H···Cl and N-H···Cl, the proportion of the interaction accounts for 19.1% of the total Hirshfeld surface, which indicates that the strong interaction is mainly caused by C-H···Cl and N-H···Cl contributions.

In Ade-Br, the intermolecular interactions between the carbon, nitrogen, and H atoms on the adenine molecule appear as red regions on the Hirshfeld surface, with bright red spots corresponding to C-H···Br and N-H···Br interactions, intermolecular interactions in 2D fingerprinting. In C-H···Br and N-H···Br, the proportion of the interaction accounts for 25.4% of the total Hirshfeld surface, which indicates that the strong interaction is mainly caused by C-H···Br and N-H···Br contributions.

### 2.3. Infrared Spectra of Compounds ***1*** and ***2***

Infrared spectroscopy is generally a fast way to analyze the active components in compounds. The compounds were weighed with dried potassium bromide in a molar ratio of 1:100, and the two were mixed and ground thoroughly and pressed into tablets; FT–IR analyses of Ade-Cl and Ade-Br were carried out in the range of 4000–400 cm^−1^.

**Figure 6:**
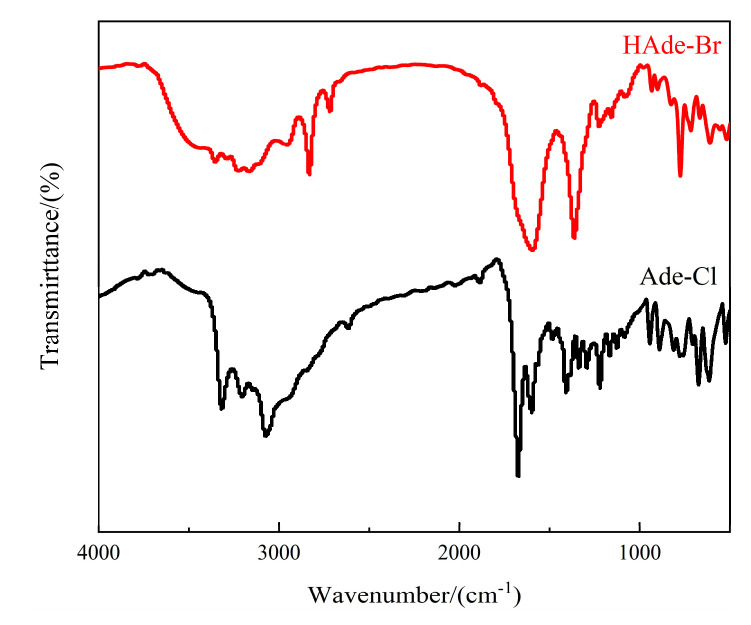
Infrared spectra of Ade-Cl and Ade-Br.

The vibrational peaks of Ade-Cl (Figure 6) were assigned as follows: the peak at 2925 cm^−1^ was assigned to the C-H stretching vibration; the peaks at 1174, 1128, and 1081 cm^−1^ were the bending vibrations in the CH plane of the ring; those at 812, 765, and 673 cm^−1^ arose from out-of-plane CH bending vibrations; the strong absorption peaks at 1392 and 1587 cm^−1^ arose from the vibration of the adenine skeleton; the peak at 3306 cm^−1^ was the telescopic vibration of the primary amine NH_2_; the peaks at 3213 and 3320 cm^−1^ were the telescopic vibrations of NH^+^; the peak at 1667 cm^−1^ arose from the telescopic vibration of the C=C double bond on the ring; and the peak 1411 cm^−1^ was attributed to the telescopic vibration of C-N. From the spectral profile, it is inferred that adenine is the main component of Ade-Cl.

Ade-Br was obtained by substituting Br atoms for the Cl atoms, and the infrared spectrum was very similar to that of Ade-Cl. The relevant vibration peaks were as follows (Figure 5): the weak peak at 2953 cm^−1^ was the C-H telescopic vibration; the peaks at 1160 and 1081 cm^−1^ were the intra-plane bending vibrations of CH on the ring; the peaks at 826, 775, and 709 cm^−1^ were the extra-plane bending vibrations of CH; the intense absorption peak at 1351 arose from the vibration of the adenine skeleton; the peak at 3348 cm^−1^ was the telescopic vibration of the primary amine (NH_2_); the peaks at 3222 and 3167 cm^−1^ were the telescopic vibrations of NH^+^; the peak at 1602 cm^−1^ was the telescopic vibration of the double bond of C=C on the ring; and the peak at 1355 cm^−1^ was the telescopic vibration of C-N. Thus, the main component of Ade-Br is adenine, as inferred from the infrared spectrum.

### 2.4. XRD Analysis of Compounds ***1*** and ***2***

To analyze and compare the purity of crystalline Ade-Cl and Ade-Br, X-ray powder diffraction data were acquired in the 2θ range of 10–50°.

**Figure 7:**
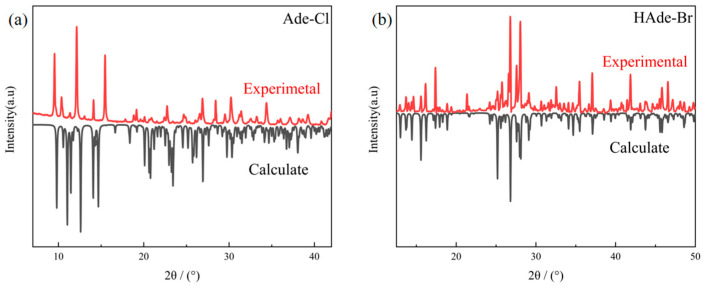
XRD powder diffraction patterns of Ade-Cl (**a**) and Ade-Br (**b**).

Figure 7a,b show the experimental and simulated XRD data for Ade-C and Ade-Br at room temperature, respectively. The experimental and simulated diffraction peak positions of the compounds were basically the same, indicating that the Ade-Cl and Ade-Br had good purity and structural homogeneity.

### 2.5. Photoluminescence Properties of Compounds ***1*** and ***2***

**Figure 8:**
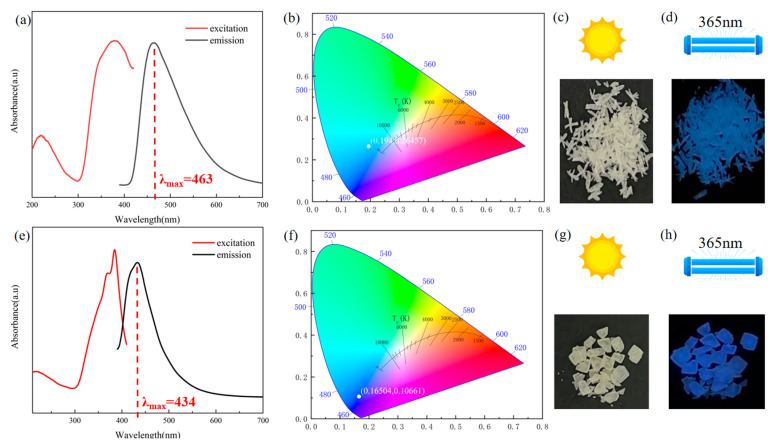
Excitation and emission spectra of Ade-Cl and Ade-Br (**a**), CIE plot (**b**), photograph of Ade-Cl under daylight lamp (**c**), photograph of Ade-Cl under 365 nm UV lamp (**d**), excitation and emission spectra of Ade-Br (**e**), CIE plot (**f**), photograph of Ade-Br under daylight lamp (**g**), photograph of Ade-Br under 365 nm UV lamp (**h**).

Compounds **1** and **2** exhibited good fluorescence properties at room temperature. Thus, excitation (PLE) and PL spectra were obtained for the analysis of the photoluminescence of solid Ade-Cl, as shown in Figure 8a. Ade-Cl absorbed UV light in the range of 200–420 nm, where the optimal excitation wavelength was 376 nm; PL mapping was performed at the optimal excitation wavelength. The broadband emission covered the entire visible range of 390–700 nm, with the strongest emission at 463 nm, the Stokes shift (Δλ = λ_em_ − λ_ex_) of Ade-Cl was 87 nm, and its photoluminescence quantum yield (PLQY) was 28.84% at room temperature. The chromaticity diagram (Figure 8b) was plotted based on the PL spectrum; the CIE coordinates were (0.194, 0.265), located in the cold blue region. As shown in Figure 8c, Ade-Cl appeared as white, transparent, rod-like crystals under natural light, while it appeared blue under 365 nm UV irradiation (Figure 8d).

Compared to solid Ade-Cl, in Ade-Br, the Br atoms replace the Cl atoms; thus, the fluorescence properties changed. The excitation (PLE) and PL spectra are shown in Figure 8e. Compound **2** absorbed UV light in the range of 210–410 nm, where the optimal excitation wavelength was 385 nm. PL mapping was performed at the optimal excitation wavelength. The broadband emission covered the entire visible range of 390–700 nm. The emission was strongest at 434 nm, the Stokes shift (Δλ = λ_em_ − λ_ex_) of Ade-Cl was 49 nm, and its photoluminescence quantum yield (PLQY) was 20.84% at room temperature. Based on the PL spectra, the chromaticity plot (Figure 8f) was obtained with corresponding CIE coordinates of (0.165, 0.107), located in the cool blue region. As shown in Figure 8g, Ade-Br appeared as colorless and transparent bulk crystals under natural light, but it appeared blue under 365 nm UV irradiation (Figure 8h). With the substitution of Br atoms for Cl atoms, the emission wavelength of Ade-Br was significantly blue-shifted, with a displacement of 29 nm toward the dark blue region.

**Figure 9:**
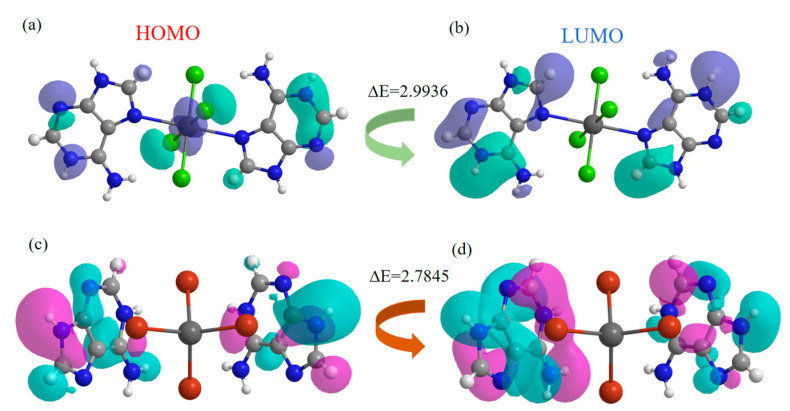
Frontier molecular orbital (FMO) diagram: HOMOs (**a**) and LUMOs (**b**) of Ade-Cl, and HOMOs (**c**) and LUMOs (**d**) of Ade-Br.

To further understand the different photoluminescence mechanisms of the two materials, energy-dependent molecular frontier orbitals (MFOs) were calculated using density functional theory (DFT) based on CIF files. The highest molecular occupied orbital (HOMO) and lowest molecular unoccupied orbital (LUMO) of the two compounds are shown. For Ade-Cl (Figure 9a,b), the HOMO of the whole molecular structure occupies the inorganic and organic parts, and the LUMO is only located in the inorganic components, with HOMO and LUMO energies of −0.1124 and 2.8812 eV, respectively, with an energy difference of 2.9936 eV. The LUMO is mainly contributed to by the pyrimidine and imidazole rings of adenine. Thus, the overlap of the aromatic parts leads to the existence of π-π interactions, and the combination of the organic and inorganic parts causes Ade-Cl to exhibit blue emission. The HOMO and LUMO of Ade-Br (Figure 9c,d) occupy both the organic and inorganic bromine atoms. The corresponding energies are −0.0328 and 2.7845 eV, respectively, and the corresponding energy difference is 2.8173 eV. The π-π interactions between the organics and the introduction of bromine atoms give them a blue emission.

The results revealed that substitution of the chlorine atoms is effective in enhancing the fluorescence emission intensity, and the introduction of bromine atoms reduces the fluorescence emission intensity and photoluminescence quantum yield, suggesting that the regular substitution of halogen atoms can provide a new avenue for developing new solid fluorescent materials.

### 2.6. Electronic Structure Calculations of Compounds ***1*** and ***2***

**Figure 10:**
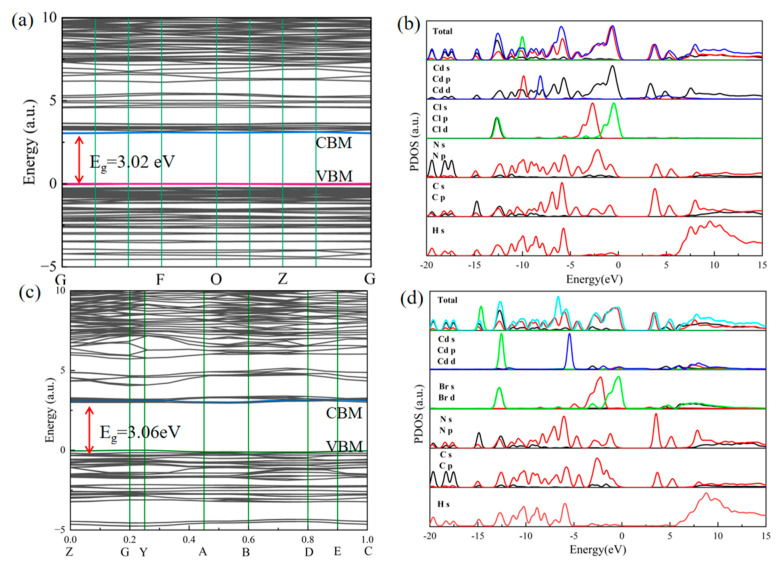
Band structure of Ade-Cl (**a**), partial density of state (DOS) of Ade-Cl (**b**), band structure of Ade-Br (**c**), partial density of state (DOS) of Ade-Br (**d**).

Organic-–inorganic hybrid materials are potential semiconductor materials. In order to further understand the semiconductor properties of these materials, the band structure and corresponding density of states (DOS) were calculated. As shown in Figure 10, the theoretical bandgaps of the two compounds are 3.02 and 3.06 eV, respectively. The CB minimum value (CBM) and valence band maximum value (VBM) were located parallel to the space orbit, indicating that they are direct-band-gap semiconductors. It can be seen from the PDOS data that the CBs of Ade-Cl and Ade-Br are mainly attributed to the inorganic component, and the inorganic part of Ade-Cl is more involved in the metal cadmium, which has a high quantum yield. The wide band gap in Ade-Br makes its quantum yield very low. Therefore, its optical properties are the result of the synergistic action of the inorganic and organic parts. The state density indicates that the electrons of the band structure are mainly contributed to by the inorganic skeleton and organic ions. The luminescence mechanism is similar to that of reported Cd-based hybrid materials [29,39,41,52].

### 2.7. Thermal Analysis of Compounds ***1*** and ***2***

**Figure 11:**
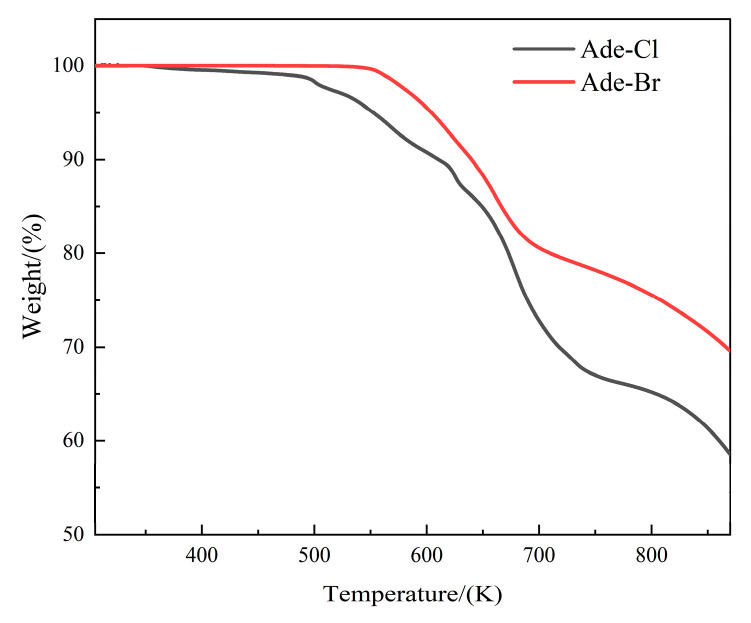
TG curves of Ade-Cl and Ade-Br.

To analyze the relationship between the components of Ade-Cl and Ade-Br and their thermal stability, thermogravimetric tests were carried out under nitrogen at a heating rate of 10 K/min over the temperature range of 300–850 K. The TG curves are shown in Figure 11, and the DTG curves are shown in Figure S5.

The Ade-Cl underwent two decomposition processes, where 341–740 K was the first stage, and the actual decomposition mass ratio was 32.4%; the loss was due to a molecule of protonated adenine cation and a chloride ion in the Ade-Cl, which was consistent with its theoretical weight loss value of 33.1%. The DTG curve showed a number of peaks. A strong and sharp peak appeared at 677 K, and the decomposition rate reached the maximum value; the residual mass was 66.9%. In the second stage at 740–867 K, the remainder continued to decompose with heat in the Ade-Cl.

Compared with the Ade-Cl, the thermal stability of the Ade-Br was significantly improved due to the substitution with Br atoms, as shown in Figure 11. The Ade-Br underwent two decomposition processes. At 300–551 K, the compound was more thermally stable and did not decompose. The first decomposition stage was 551–698 K, with a mass loss of 19.70%, corresponding to the loss of [C_5_N_5_H_6_]^+^, which is the protonated adenine cation in Ade-Br. This mass was approximately the same as the theoretical weight loss value of 19.30%. The DTG curve showed a short and wide peak and a strong and sharp peak at 660 K, and the decomposition rate reached the maximum value. The residual mass was 80.30%. When the temperature reached 715 K, the compound did not decompose, but it continued to decompose when the temperature was further increased. In the second stage at 715–871 K, the decomposition was presumed to involve [CdBr_4_]^2−^.

As shown in Figure 11, the initial thermal decomposition temperature of Ade-Cl was 341 K, while that of Ade-Br was 551 K. The initial decomposition temperature of Ade-Br increased by ~210 K compared with that of Ade-Cl, which indicates that the Ade-Cl underwent stronger thermal motion. This difference may be due to the introduction of bromine atoms, which make Ade-Br more thermally stable.

### 2.8. Dielectric Properties of Compounds ***1*** and ***2***

**Figure 12:**
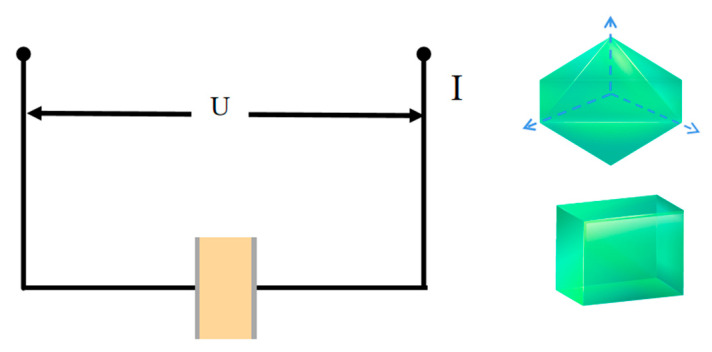
Image of a schematic illustration of the dielectric measurement mechanism for the compounds along the bc plane.

The crystal structures of the molecules undergo telescopic deformation and torsion, which can cause changes in the electrical properties of the compounds. Ade-Cl, with its small crystal size, was pressed in order to test its dielectric properties. The Ade-Cl crystal size is small, and an electrode was made after pressing it; Ade-Br in the *a*, *b*, and *c* axial directions was made into four capacitors using conductive silver glue and copper wire. Figure 12 shows a schematic illustration of the dielectric measurement mechanism.

**Figure 13:**
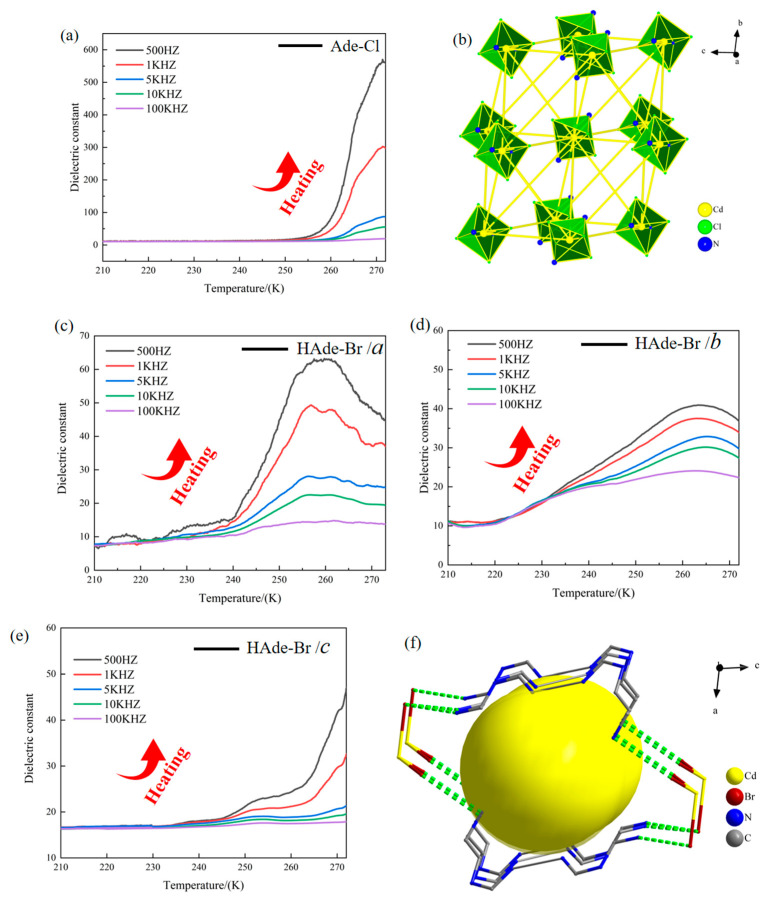
(**a**) Dielectric constant of Ade-Cl; (**b**) three-dimensional frame structure arrangement of Ade-Cl; (**c**) dielectric anomaly of Ade-Br in the *a*-axis; (**d**) dielectric anomaly of Ade-Br in the *b*-axis; (**e**) dielectric anomaly of Ade-Br in the *c*-axis; (**f**) three-dimensional frame structure arrangement of Ade-Br.

Variable-temperature–variable-frequency dielectric tests were conducted in the temperature range of 210–275 K and frequency range from 500 Hz to 100 KHz. Both Ade-Cl and Ade-Br exhibited similar trends in their dielectric changes during the heating process. The dielectric constant decreased as the electric field increased, reaching a peak and then gradually decreasing at higher temperatures. Figure 13a shows the dielectric constant of Ade-Cl during the low-temperature heating process. Abnormal dielectric behavior was observed around 255 K, with a sudden increase in the dielectric constant after 258 K, reaching a peak at 271 K.

Figure 13c presents the dielectric constant of Ade-Br along the a-axis during heating in the low-temperature region. An abnormal dielectric phenomenon was observed around 223 K, with a sudden increase in the dielectric constant after 235 K, reaching a peak at 260 K. The dielectric constant slowly decreased as the temperature increased. Figure 13d shows the dielectric constant of Ade-Br along the b-axis with heating in the low-temperature region. An abnormal dielectric phenomenon was observed around 256 K, with a sudden increase in the dielectric constant after 260 K, reaching a peak at 270 K. Figure 13e shows the dielectric constant of Ade-Br along the c-axis during low-temperature heating. A relatively small and anomalous step-like dielectric peak was observed around 235 K, followed by a second anomalous step-like dielectric peak at 246 K. With increasing the temperature, the change in the dielectric constant was more pronounced, increasing sharply after 263 K and reaching a peak at 270 K.

The analytical data show that both Ade-Cl and Ade-Br exhibited dielectric anomalies at 210–275 K. Based on the structures of the compounds, it is hypothesized that the dielectric anomalies of Ade-Cl are attributable to the deformation of the three-dimensional framework and the telescopic and torsional deformation of [CdCl_4_] with temperature, as shown in Figure 13b, which ultimately resulted in the generation of the anomalous dielectric peaks. For Ade-Br, the dielectric anomalies were found in the three axial directions. The anisotropic dielectric anomalies were attributed to the telescopic and torsional deformation of the hydrogen bonds in the three-dimensional hydrogen-bonded framework structure as well as the telescoping of the three-dimensional framework cavities, as shown in Figure 13f.

### 2.9. Electrochemical Properties of Compounds ***1*** and ***2***

**Figure 14:**
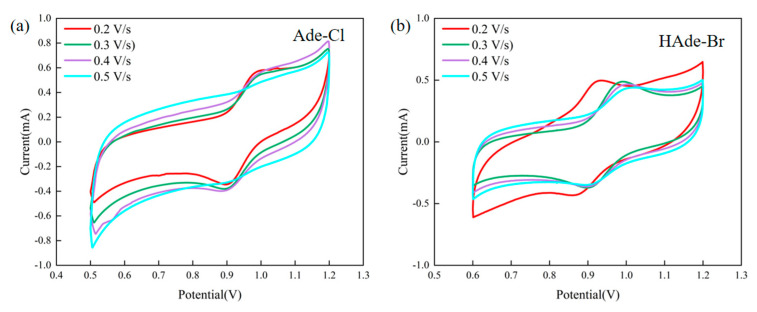
Cyclic voltammetry curves of Ade-Cl (**a**) and Ade-Br (**b**).

The electrochemical properties of organic–inorganic hybrid materials are closely related to their thermochemistry, molecular motions, structural properties, etc. To further investigate the electrical properties of Ade-Cl [53,54], cyclic voltammetry (CV) data were acquired using a three-electrode system (glassy carbon electrode, auxiliary electrode, and reference electrode) in a mixture of 0.1 mol/L H_2_SO_4_ and 0.5 mol/L Na_2_SO_4_. Figure 14a shows the cyclic voltammetry curves of Ade-Cl in the voltage range of 0.5–1.2 V at sweep speeds of 0.2, 0.3, 0.4, and 0.5 V/s. A reduction peak was also observed within the negative potential region, indicating that Ade-Cl exists in an oxidized state. The widest separation between the anodic and cathodic peak potentials was observed at the sweep rate of 0.2 V/s, with *E*_pc_ = 0.980 V and *I*_pc_ = 0.565 × 10^−5^ A. In the opposite direction, *E*_pa_ = 0.901 V and *I*_pa_ = −0.340 × 10^−5^ A. The standard electrode potential is half of the sum of the two peaks according to the following equation: *E_θ_ =* (*E*pa-*E*pc)/2, where *E_θ_* = 0.980 V. According to the equation, Δ*E*p = *E*pa-*E*pc = 59/n; Δ*E*p = 0.085 V, where Δ*E*p = 59/*n* = 0.084. *I*_pa_*/I*_pc_ = 0.85, which is close to unity, indicating that the electrochemical reaction is reversible [55,56].

Figure 10b shows the cyclic voltammograms of Ade-Br in the voltage range of 0.6–1.2 V at sweep speeds of 0.2, 0.3, 0.4, and 0.5 V/s. A reduction peak was observed in the negative potential region, indicating an oxidized state of Ade-Br. The widest separation between the anodic and cathodic peak potentials was observed at the sweep rate of 0.2 V/s, with *E*_pc_ = 0.926 V and *I*_pc_ = 0.493 × 10^−5^ A. In the opposite direction *E*_pa_ = 0.869 V and *I*_pa_ = −0.435 × 10^−5^ A. The standard electrode potential is half of the sum of the two peaks according to the equation *E_θ_ =* (*E*pa − *E*pc)/2. According to this equation, Δ*E*p = *E*pa − *E*pc = 59/n, *E_θ_* = 0.847 V, and Δ*E*p = 0.057 V, where Δ*E*p = 59/*n* = 0.056. *I*_pa_*/I*_pc_ = 0.88, which is close to 1, indicating that the electrochemical reaction is reversible, demonstrating that both compounds have good electrocatalytic properties [57,58,59].

## 3. Materials and Methods

### 3.1. Reagents and Instruments

Reagents: Adenine, CdCl_2_, deionized water, hydrochloric acid, and hydrobromic acid were commercially available in analytical purity.

Instruments: Infrared spectrometer (Thermo Fisher Nicolet iS5, Waltham, MA, USA), single-crystal X-ray diffractometer (Bruker Smart Apex Ⅱ tester, Bruker, Karlsruhe, Germany), powder X-ray diffractometer (Germany Bruker D2 PHASER, Bruker, Karlsruhe, Germany), thermogravimetric analyzer (Q50 Thermogravimetric Analysis TA, New Castle, DE, UAS), dielectric electrolytic properties tester (Tonghui TH2828A, New Castle, DE, USA), and CHI 700E electrochemical workstation (Shanghai Chenhua Instrument Co., Ltd., Shanghai, China).

### 3.2. Synthesis of Compounds

**Figure 15:**
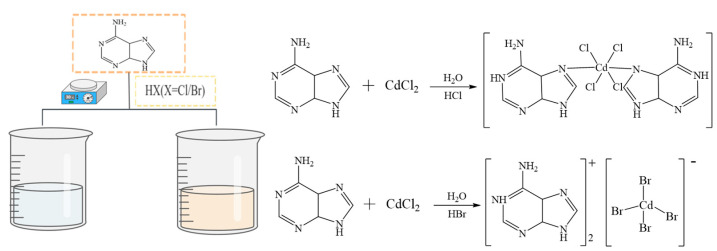
Schematic of synthesis of Ade-Cl and Ade-Br.

Adenine (0.147 g, 0.20 mmol) was weighed and added to 10 mL of distilled water; some of the adenine did not dissolve. CdCl_2_ (0.201 g, 0.217 mmol) was weighed and completely dissolved in 10 mL of distilled water and then added to 1 mL of HCl solution. The obtained CdCl_2_ aqueous solution was slowly added dropwise to the incompletely dissolved adenine aqueous solution. Consequently, the adenine was completely dissolved. A clear and transparent mixed solution of adenine and CdCl_2_ in a molar ratio of 1:1 was obtained and placed in a room-temperature environment, and a transparent, strip-crystal material (Ade-Cl) (**1**) was obtained after seven days using the room-temperature evaporation method. The synthesis route is shown in Figure 15.

Using the same method, hydrochloric acid was replaced with hydrobromic acid, as shown in Figure 14, to obtain transparent lamellar crystals (Ade-Br) as follows: adenine (0.147 g, 0.20 mmol) was weighed and added to 10 mL of distilled water; some of the adenine did not dissolve. CdCl_2_ (0.401 g, 0.434 mmol) was also weighed and completely dissolved in 10 mL of distilled water, followed by the addition of 1 mL HBr solution; the CdCl_2_ aqueous solution was slowly added dropwise to the incompletely dissolved adenine aqueous solution. The resulting solution was light brown and transparent, with a molar ratio of adenine to cadmium chloride of 1:2. The solution was left to stand at room temperature, and after seven days of evaporation, transparent block-shaped crystals (Ade-Br) were obtained.

### 3.3. Crystal Structure Determination

An Ade-Cl crystal (0.15 mm × 0.13 mm × 0.12 mm) with an unadulterated surface and suitable size was selected; graphite-monochromatized Mo-Kα radiation (λ = 0.71073 nm) was used as the radiation source for the XRD analysis, whereas for Ade-Br (0.13 mm × 0.12 mm × 0.11 mm), graphite-monochromatized Cu-Kα radiation was used (λ = 1.54184 nm). The sample was placed on a single-crystal X-ray diffractometer, and diffraction data were collected at low temperature (100 K) and room temperature (293 K), respectively. The crystal structure was resolved by the direct method through the SHELXL⁃97 program and refined using the full matrix method based on *F*^2^. All non-hydrogen atoms were corrected using the anisotropy correction, and the positions of the hydrogen atoms in the water molecules were confirmed by means of the electron cloud density. The structural data and refinement parameters of Ade-Cl and Ade-Br are shown in Table 1.

## 4. Conclusions

Two new organic–inorganic hybrid crystalline compounds, Ade-Cl (**1**) and Ade-Br (**2**), were obtained by a room-temperature evaporation method using adenine and cadmium chloride as the raw materials, hydrochloric acid and hydrobromic acid as the halogen sources, and water as a solvent, and their structures and properties were analyzed. As confirmed by single-crystal X-ray diffraction, Ade-Cl and Ade-Br, which differed in terms of replacement of the Cl atoms by Br atoms, crystallized in a triclinic crystal system with the *P*-1 space group and a monoclinic crystal system with the *P*2_1_/m space group, respectively. The cell parameters all changed with temperature. It is noteworthy that the nitrogen atom in the Ade-Cl mesoadenine is connected to the metal ion through a Cd-N bond to form a monolithic unit, whereas Ade-Br is an ionic complex and forms a monolithic unit through a non-covalent bond. Both Ade-Cl and Ade-Br form a three-dimensional framework structure under the action of hydrogen bonding, which provides sufficient space for molecular motion, intermolecular and intermolecular vibrations, and stretching deformations, leading to changes in the optical, electrical, and thermal properties of the materials. The stability of Ade-Br is much higher than that of Ade-Cl due to halogen substitution, whereas substitution with the bromine atoms leads to a shift in the optimal PL emission wavelength by 29 nm toward the deep blue region. This change decreases the intensity of the fluorescence emission, attributed to electronic transition between the Cl(3p), Cd(4p), and Cd(5s) orbitals. Cyclic voltammetry confirmed the good reversibility of the electrolytic process and the excellent electrocatalytic properties of the materials, which can provide more possibilities for the future synthesis of organic–inorganic hybrid multifunctional coupled materials with PL, dielectric anisotropy, and electrocatalytic properties.

## Figures and Tables

**Table 1:** Crystallographic parameters of Ade-Cl and Ade-Br.

Compounds	Ade-Cl		Ade-Br	
Temperature	100 K	293 K	100 K	293 K
Chemical formula	C_5_H_5_Cd_0_._5_Cl_2_N_5_	C_5_H_5_Cd_0_._5_Cl_2_N_5_	C_10_H_12_N_10_CdBr_4_	C_10_H_12_N_10_CdBr_4_
Formula weight	526.50	526.50	704.34	704.34
Crystal size (mm)^3^	0.16 × 0.13 × 0.12	0.15 × 0.13 × 0.12	0.13 × 0.12 × 0.11	0.13 × 0.12 × 0.11
Crystal system	triclinic	triclinic	monoclinic	monoclinic
Space group	*P-1*	*P-1*	*P*2_1_/m	*P*2_1_/m
a (Å)	0.74465 (6)	0.75040 (8)	0.645740 (10)	0.650350 (10)
b (Å)	0.84468 (6)	0.85271 (9)	2.05480 (3)	2.06785 (3)
c (Å)	1.46159 (11)	1.47168 (11)	0.685480 (10)	0.689280 (10)
α (°)	93.221 (6)	92.638 (7)	90	90
β (°)	103.718 (7)	104.088 (8)	99.4100 (10)	99.1640 (10)
γ (°)	111.405 (6)	111.840 (10)	90	90
V (Å)^3^	821.08 (11)	838.00 (15)	897.30 (2)	915.13 (2)
Z	4	4	2	2
D_calc_ (g-cm)^−1^	262.24	262.24	2.607	2.556
F (000)	512.0	512.0	660.0	660.0
μ (mm)^−1^	2.000	1.959	20.358	19.961
Measured 2 range (°)	5.246–49.97	5.206–49.998	13.786–143.658	13.7–133.13
R_int_	0.0289	0.0295	0.0209	0.0246
R (I > 2 (I)) ^[a]^	0.0438	0.0408	0.0388	0.0381
_W_R (all data) ^[b]^	0.1273	0.1031	0.1059	0.1074
GOF	1.117	1.102	1.058	1.086
CCDC NO.	2,326,393	2,326,394	2,326,404	2,326,405

$[a]:R=\sum (\left|F_{o}\right|-\left|F_{c}\right|)/\sum \left|F_{o}\right|$, $[b]:R_{w}^{2}=\sum_{w}{\left(F_{o}^{2}-F_{c}^{2}\right)}^{2}/\sum_{w}{\left(F_{o}^{2}\right)}^{2}$.

## Data Availability

The data presented in this study are available on request from the corresponding author.

## Supplementary Materials

The following supporting information can be downloaded at: https://www.mdpi.com/1420-3049/29/12/2773/s1, Table S1: Selected bond lengths (Å) and bond angles (°) of Ade-Cl; Table S2: Selected bond lengths (nm) and bond angles (°) of Ade-Br; Table S3: Hydrogen bond parameters of Ade-Cl; Table S4: Hydrogen bond parameters of Ade-Br; Figure S1: Symmetry structure diagram of Ade-Cl at 100 K; Figure S2: Arrangement diagram of Ade-Cl in the *c*-axis at 100 K; Figure S3: Arrangement diagram of Ade-Br in the *b*-axis at 100 K; Figure S4: Stacking diagram of Ade-Br in the *c*-axis at 100 K; Figure S5: DTG curves of Ade-Cl and Ade-Br.
