# Supplementary material for: Tunability of Photovoltaic Functions via Halogen Substitution [(Ade)2 CdX4](X = Cl, Br): A Class of Three-Dimensional Organic–Inorganic Hybrid Materials
Source: Molecules. 2024 Jun 11;29(12):2773. doi: 10.3390/molecules29122773 (PMC11487418; doi:10.3390/molecules29122773)
Supplement: Supplementary file 1 [file molecules-29-02773-s001.zip › molecules-2971609-supplementary.pdf]

Tunability of photovoltaic functions via halogen substitution [(Ade)$_2$ CdX$_4$](X = Cl, Br): A class of three-dimensional organic-inorganic hybrid materials

Mei-xia Lv [a], Hong-zhi Hu [a,b] , Abuduheni • Adila [a], Yi-bo Yan [a,b], Yang Liu [a,b,c,*], Zun-qi Liu[a,b,c,*]

[a] *Chemistry and Chemical Engineering College Xinjiang Agricultural University;*
[b]*Xinjiang Sub-Center National Engineering Research Center of Novel Equipment for Polymer Processing;*
[c]*Xinjiang Key Laboratory of Agricultural Chemistry and Biomaterials, Urumqi 830052, Xinjiang*

Table S1. Selected bond lengths (nm) and bond angles (°) of Ade-Cl

			LT		
Cd1-Cl1	0.25708(15)	Cd2-N10	0.2527(5)	C3-C4	0.1387(9)
Cd1-Cl2	0.25698(15)	N1-C2	0.1311(8)	N7-C9	0.1367(8)
Cd1-N5	0.2509(5)	N2-C1	0.356(8)	N9-C8	0.1363(8)
Cd2-Cl3	0.25363(15)	N3-C1	0.1313(8)	N10-C7	0.1381(8)
Cd2-Cl4	0.26106(15)	N3-C4	0.1362(8)	C7-C8	0.1431(9)
Cl1-Cd1-Cl1^1	180.00(7)	N5-Cd1-Cl2	89.29(12)	N10-Cd2-Cl3	89.13(12)
Cl2-Cd1-Cl1	90.60(5)	N5-Cd1-Cl2^1	90.71(12)	N10^2-Cd2-Cl3	90.87(12)
Cl2-Cd1-Cl1^1	89.40(5)	C3-N5-Cd1	129.9(4)	N10-Cd2-Cl4	86.55(12)
N5-Cd1-Cl1^1	85.86(13)	C5-N5-Cd1	113.5(4)	N10-Cd2-Cl4^2	86.54(12)
N5-Cd1-Cl1	94.14(13)	Cl3-Cd2-Cl4	90.32(5)	C6-N10-Cd2	112.8(4)
			RT		
Cd1-Cl1	0.2575(13)	Cd2-N10	0.2554(4)	C3-C4	0.1353(6)
Cd1-Cl2	0.2569(13)	N1-C2	0.1315(6)	N7-C9	0.1356(7)
Cd1-N5	0.2532(4)	N2-C1	0.1354(7)	N9-C8	0.1359(6)
Cd2-Cl3	0.2536(12)	N3-C1	0.1304(7)	N10-C7	0.1384(6)
Cd2-Cl4	0.2614(13)	N3-C4	0.1353(6)	C7-C8	0.1384(7)
Cl1-Cd1-Cl1^1	180.00(6)	N5-Cd1-Cl2	89.52(10)	N10-Cd2-Cl3	90.52(9)
Cl2-Cd1-Cl1	89.40(4)	N5-Cd1-Cl2^1	90.48(10)	N10^2-Cd2-Cl3	89.49(9)
Cl2-Cd1-Cl1^1	90.60(4)	C3-N5-Cd1	129.7(3)	N10-Cd2-Cl4	93.41(9)

N5-Cd1-Cl1[1]	93.75(10)	C5-N5-Cd1	113.9(3)	N10-Cd2-Cl4[2]	86.59(9)

Table S2. Selected bond lengths (nm) and bond angles (°) of Ade-Br

LT					
Cd-Br1	0.25769(10)	N1-C1	0.1311(7)	N3-C5	0.1306(8)
Cd-Br2	0.25780(7)	N2-C1	0.1372(7)	N4-C3	0.1367(7)
Cd-Br2[1]	0.25780(7)	N2-C5	0.1356(7)	N5-C2	0.1378(7)
Cd-Br3	0.25762(10)	N3-C3	0.1358(7)	N5-C4	0.1321(8)
Br1-Cd1-Br2	104.34(2)	C1-N2-C5	124.0(5)	N1-C1-C2	124.7(5)
Br1-Cd1-Br3	110.17(3)	C5-N3-C3	112.4(5)	N5-C2-C1	130.5(5)
Br3-Cd1-Br2	110.97(2)	C3-N4-C4	106.1(5)	N1-C1-N2	121.8(5)
Br2[1]-Cd1-Br2	115.53(4)	C4-N5-C2	103.0(5)	N3-C3-N4	127.4(5)
RT					
Cd-Br1	0.25770(11)	N1-C1	0.1304(8)	N3-C5	0.1298(8)
Cd-Br2	0.25786(7)	N2-C1	0.1357(7)	N4-C3	0.1356(7)
Cd-Br2[1]	0.25786(7)	N2-C5	0.1366(8)	N5-C2	0.1394(7)
Cd-Br3	0.25778(10)	N3-C3	0.1355(7)	N5-C4	0.1297(8)
Br1-Cd1-Br2	104.41(2)	C1-N2-C5	124.1(5)	N1-C1-C2	124.6(5)
Br1-Cd1-Br3	110.14(4)	C5-N3-C3	112.4(5)	N5-C2-C1	130.6(5)
Br3-Cd1-Br2	110.86(2)	C3-N4-C4	106.1(5)	N1-C1-N2	122.3(5)
Br2[1]-Cd1-Br2	115.68(4)	C4-N5-C2	103.4(5)	N3-C3-N4	127.9(5)

Table S3. Hydrogen bond parameters of Ade-Cl

D-H···A	d(D-H)nm	d(H···A)nm	d(D···A)nm	∠D-HA(°)
LT				
N1-H1A···Cl1	0.08803	0.25447	0.33070	145.349
N1-H1B···Cl2	0.08799	0.22765	0.31372	165.883
N8-H8B···Cl3	0.08804	0.22632	0.31369	171.678
N4-H4···Cl4	0.08800	0.23526	0.31699	154.580
C5-H5···Cl3	0.09498	0.28271	0.33736	117.586
C10-H10···Cl4	0.09503	0.30672	0.34337	104.703
RT				
N1-H1A···Cl1	0.08602	0.25843	0.33273	145.215

N1-H1B···Cl2	0.08602	0.23048	0.31471	166.291
N8-H8···Cl3	0.08586	0.22988	0.31515	171.488
N4-H4···Cl4	0.08603	0.23854	0.31880	155.431
C5-H5···Cl3	0.09296	0.28589	0.34099	119.164
C10-H10···Cl4	0.09300	0.31120	0.34792	105.588

Table S4. Hydrogen bond parameters of Ade-Br

D-H···A	d(D-H)nm	d(H···A)nm	d(D···A)nm	∠D-HA(°)
		LT		
C5-H5···Br2	0.09503	0.27340	0.35302	141.803
N1-H1A···Br1	0.08797	0.27098	0.33554	131.222
N2-H2A···Br2	0.08804	0.28474	0.34542	127.454
N4-H4···N3	0.08803	0.20752	0.29325	163.343
		RT		
C5-H5···Br2	0.09297	0.27828	0.35639	142.289
N1-H1A···Br1	0.08599	0.27427	0.33760	131.660
N2-H2A···Br2	0.08602	0.28759	0.34789	128.675
N4-H4···N3	0.08598	0.21111	0.29469	163.916

Table S1-4 shows the main bond lengths bond angles and hydrogen bonding parameters of Ade-Cl and HAde-Br. The main hydrogen bonds are N-H···Cl, C-H···Cl, N-H···Br, and C-H···Br. As the temperature increases, the hydrogen bond lengths are telescopically deformed and the hydrogen bond angles are twisted, which results in the deformation of one-, two-, and three-dimensional structures under the interactions of hydrogen bonding, which ultimately translates into the photovoltaic properties of the materials.

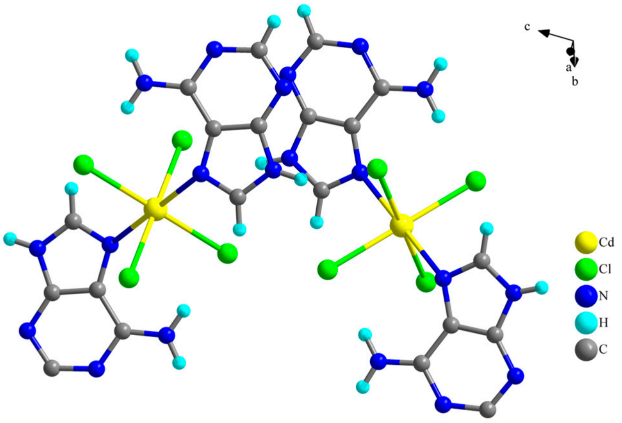

Figure S1. symmetry structure diagram of Ade-Cl at100 K.

The figure S1 shows the symmetrically operated structure of Ade-Cl with four molecules of adenine and two molecules of $[CdCl_4]_{0.5}$ linked by Cd-N bonds to form a center-pair formation.

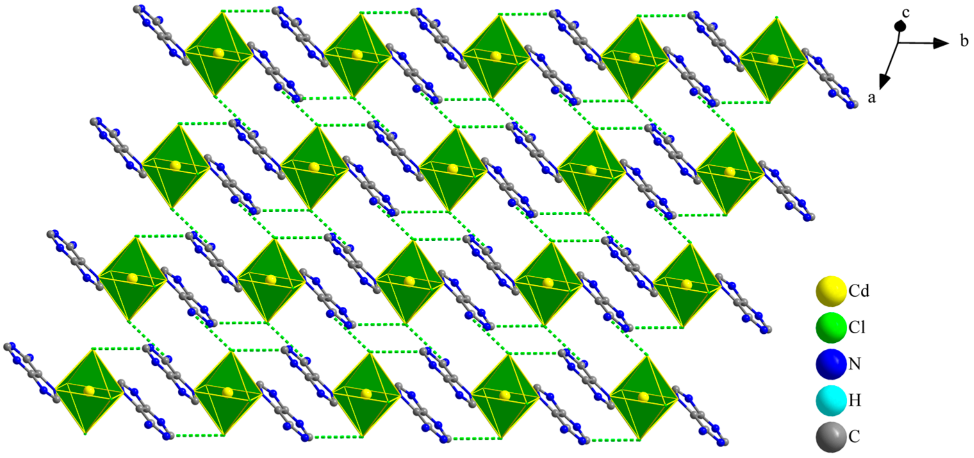

Figure S2. arrangement diagram of Ade-Cl in the *c*-axis at 100 K.

The figure S2 shows a arrangement of Ade-Cl in the low-temperature ab-plane, forming an organic-metal-organic "sandwich" structure, with intermolecular hydrogen bonding forming a matrix of hydrogen bonds in the plane, growing along the c-axis, and with the organic matter staggered up and down.

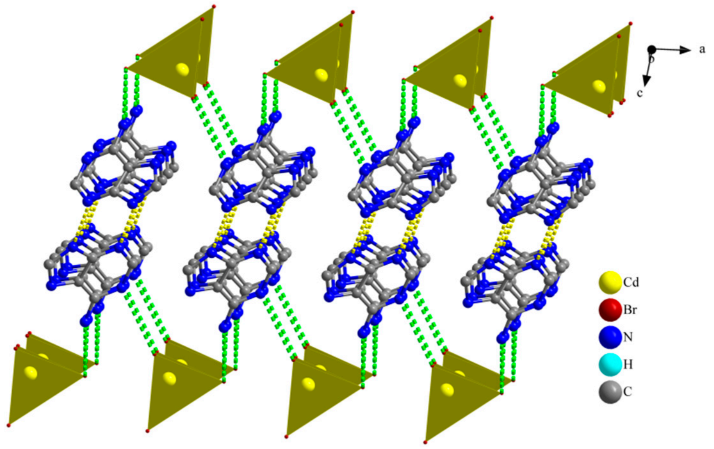

Figure S3. Arrangement diagram of Ade-Br in the *b*-axis at 100 K.

The figure S3 shows the arrangement of HAde-Br in the *b*-axis direction with cadmium atoms as vertices, with the $[CdBr_4]^{2-}$metal metal framework and the organic layer neatly aligned in the ac-plane, forming a whole under hydrogen-bonding interactions.

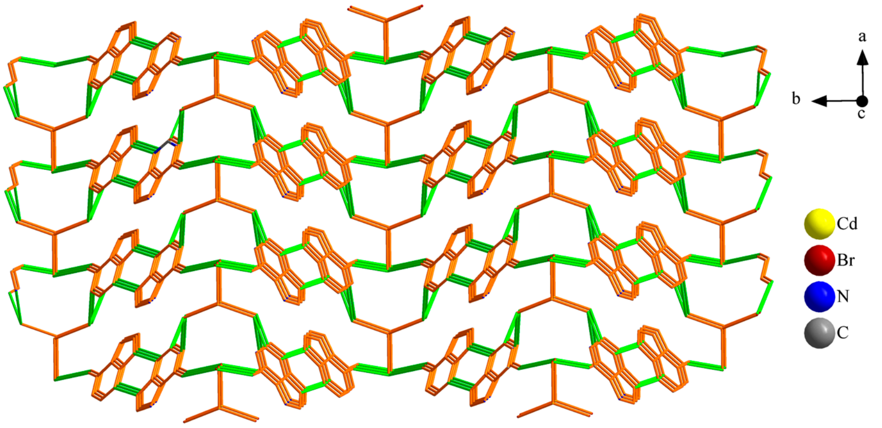

Figure S4. Stacking diagram of Ade-Br in the *c*-axis at 100 K.

The figure S4 shows the arrangement of HAde-Br in the *c*-axis direction, omitting hydrogen atoms and some atoms, the $[CdBr_4]^{2-}$metal metal framework and the adenine cation are connected by hydrogen bonding to form a framework similar to a "flower", and the adenine cation is closely connected by hydrogen bonding between two by two and is neatly arranged in the *ab*-plane.

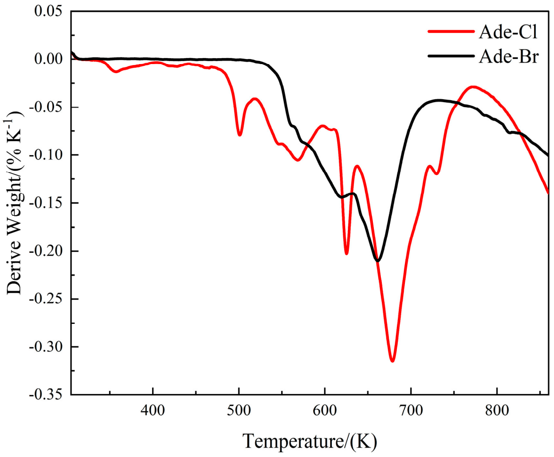

Figure S5. DTG curves of Ade-Cl and Ade-Br.

Ade-Cl showed a small and broad absorption peak in the DTG curve at 300-543 K, and a short and broad, a short and sharp and a strong and sharp absorption peak in the DTG curve at 543-737 K. Ade-Br showed no change in the DTG curve at 300-551 K, and a short and broad and a strong and sharp absorption peak in the DTG curve at 551-698 K. The DTG curves were not changed at 300-551 K, and the DTG curves showed a short and broad and a strong and sharp absorption peak at 551-698 K. The DTG curves showed the following patterns.
